# Supplementary material for: Anomalous anti-Kasha excited-state luminescence from symmetry-breaking heterogeneous carbon bisnanohoops
Source: Nat Commun. 2024 Mar 27;15:2684. doi: 10.1038/s41467-024-46848-x (PMC10973529; doi:10.1038/s41467-024-46848-x)
Supplement: Supplementary file 1 — Supplementary Information [file 41467_2024_46848_MOESM1_ESM.pdf]

Supplementary Information

Anomalous anti-Kasha excited-state luminescence from symmetry-breaking heterogeneous carbon bisnanohoops

Xinyu Zhang,[†1] Cheng Chen,[†2] Wen Zhang,[1] Nan Yin,[1] Bing Yuan,[1] Guilin Zhuang,[3] Xiao-Ye Wang,[2] Pingwu Du[1]*

[1] Hefei National Research Center for Physical Sciences at the Microscale, Key Laboratory of Precision and Intelligent Chemistry, Department of Materials Science and Engineering, University of Science and Technology of China, 96 Jinzhai Road, Hefei, Anhui Province, 230026, China

[2] State Key Laboratory of Elemento-Organic Chemistry, College of Chemistry, Nankai University, Tianjin, 300071, China

[3] College of Chemical Engineering, Zhejiang University of Technology, 18 Chaowang Road, Hangzhou, Zhejiang Province, 310032, China

[†]These authors contributed equally to this work

*Corresponding authors: dupingwu@ustc.edu.cn

Tel/Fax: 86-551-63606207

Supplementary Methods

Physical Characterizations of Compounds

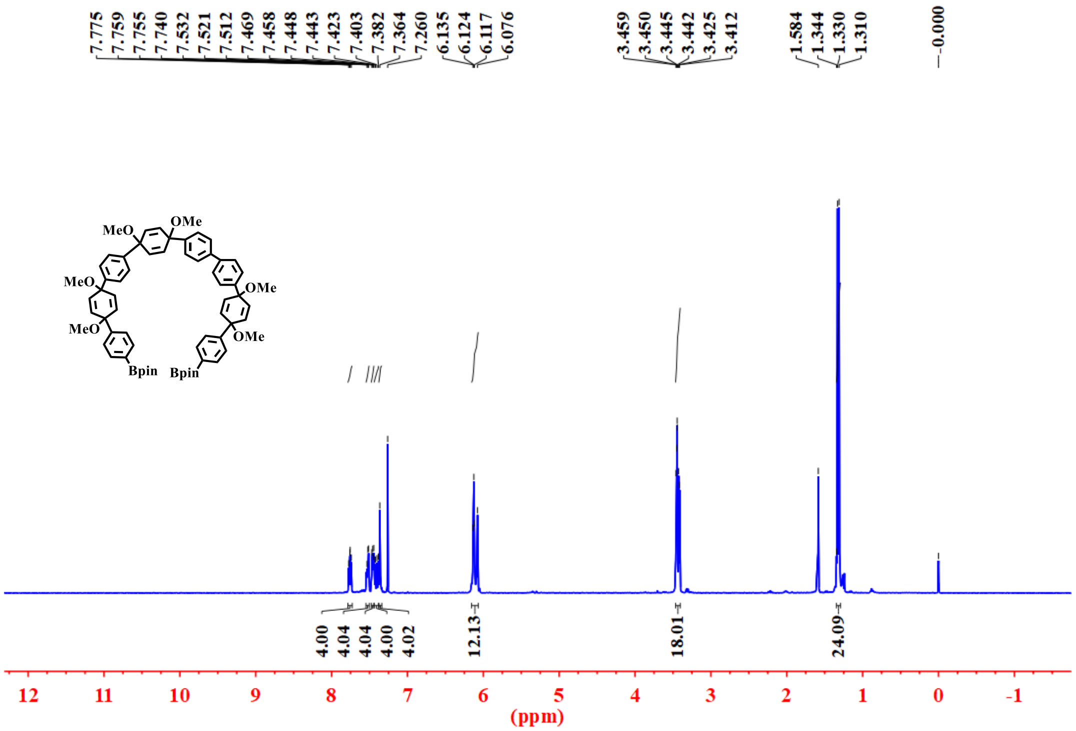

Supplementary Figure 1. ^{1}H NMR spectrum of compound **1** (400 MHz, $CDCl_3$).

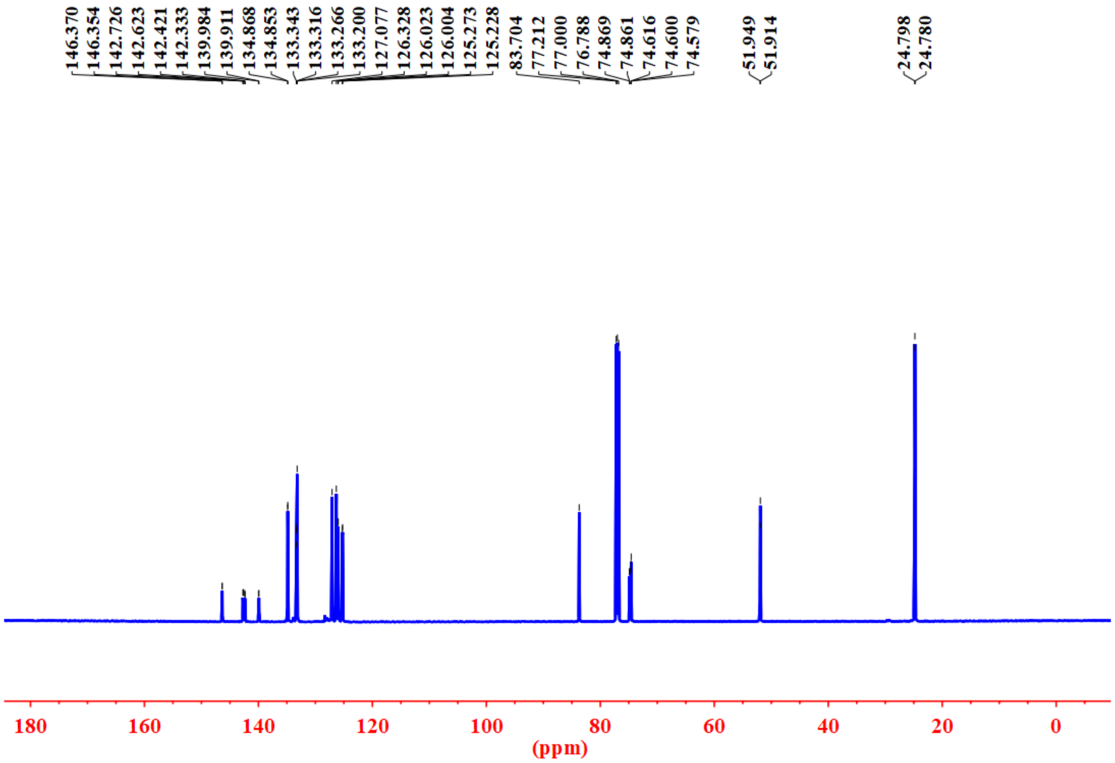

Supplementary Figure 2. ^{13}C NMR spectrum of compound **1** (100 MHz, $CDCl_3$).

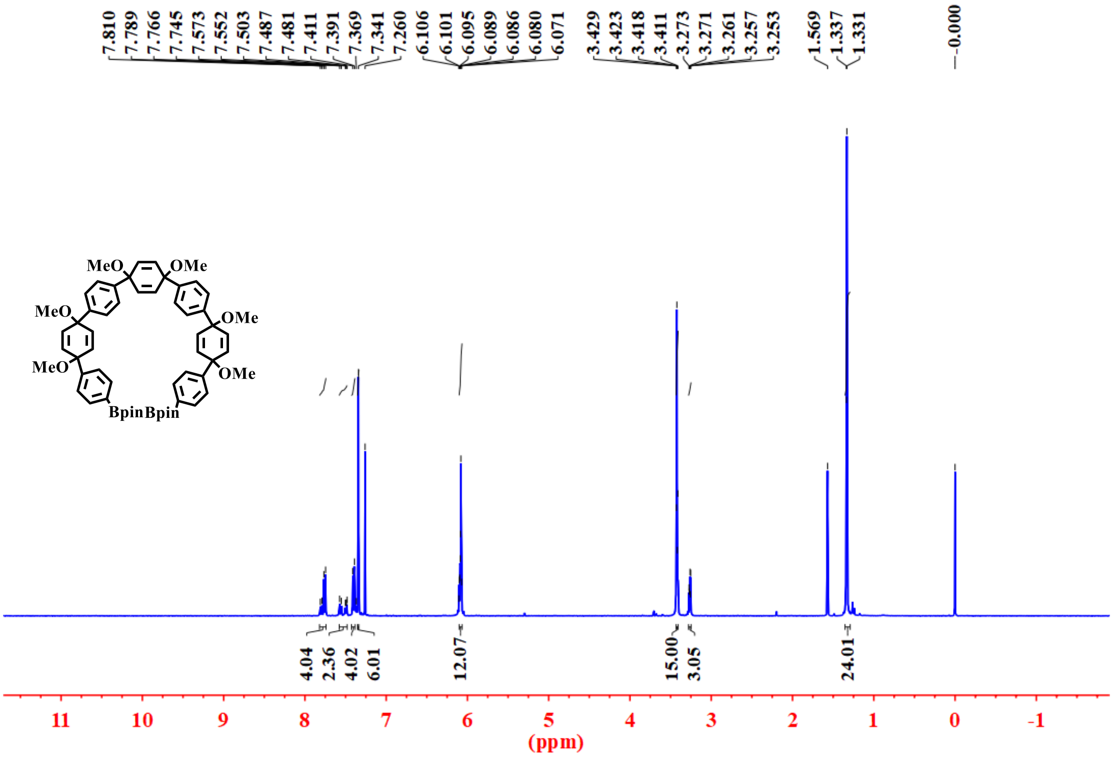

Supplementary Figure 3. ^{1}H NMR spectrum of compound **2** (400 MHz, $CDCl_3$).

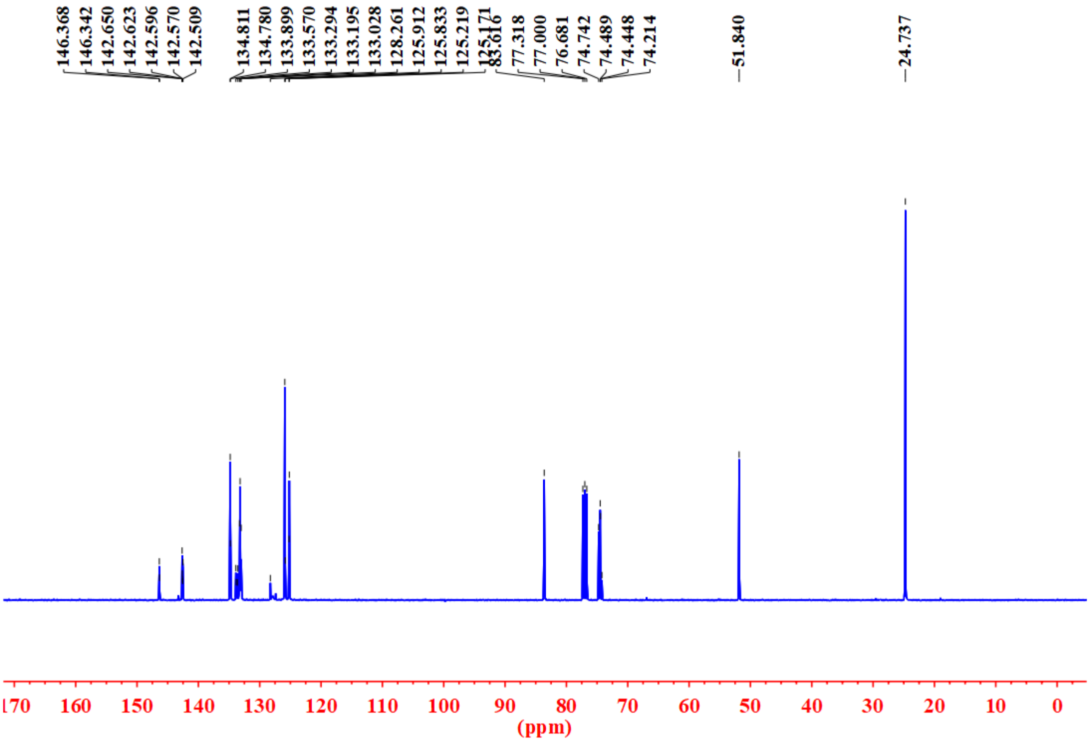

Supplementary Figure 4. ^{13}C NMR spectrum of compound **2** (100 MHz, $CDCl_3$).

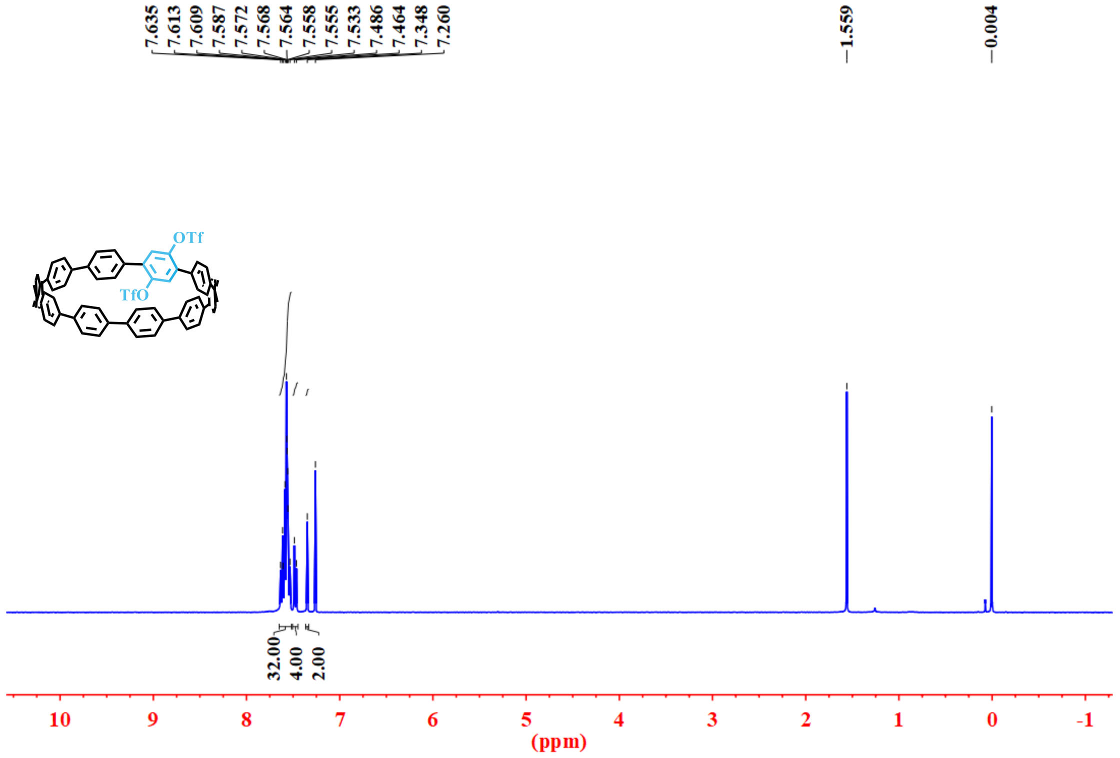

Supplementary Figure 5. ^{1}H NMR spectrum of compound **3** (400 MHz, $CDCl_3$).

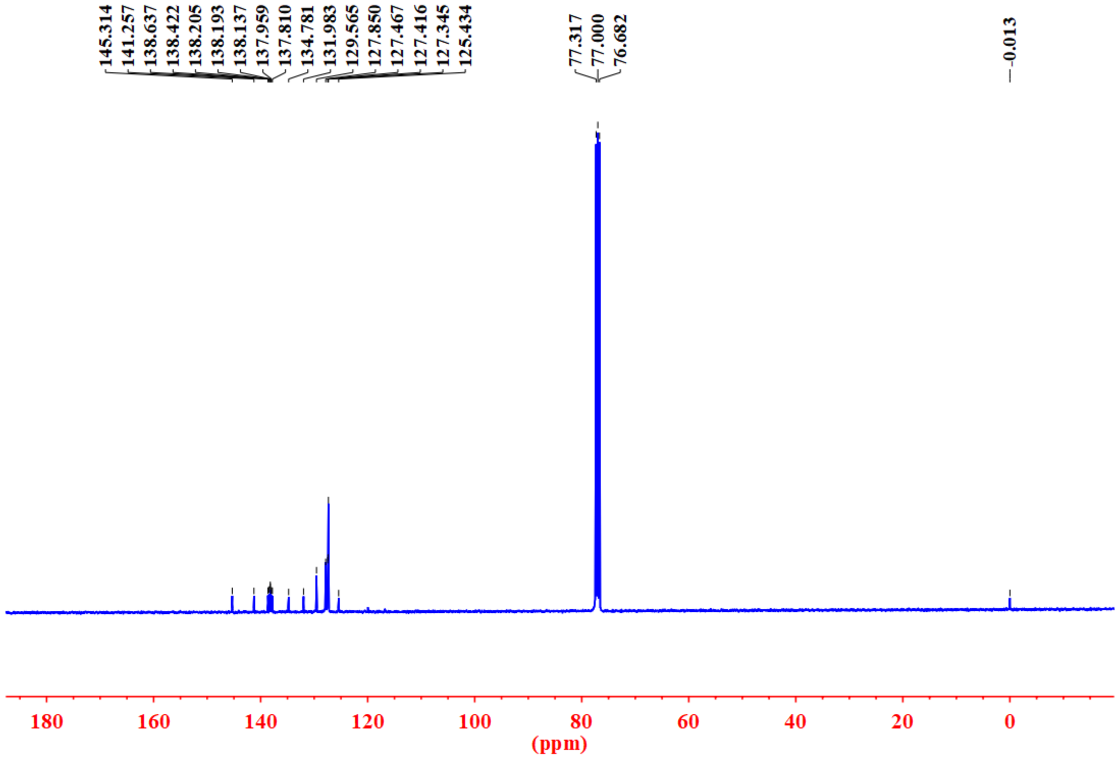

Supplementary Figure 6. ^{13}C NMR spectrum of compound **3** (100 MHz, $CDCl_3$).

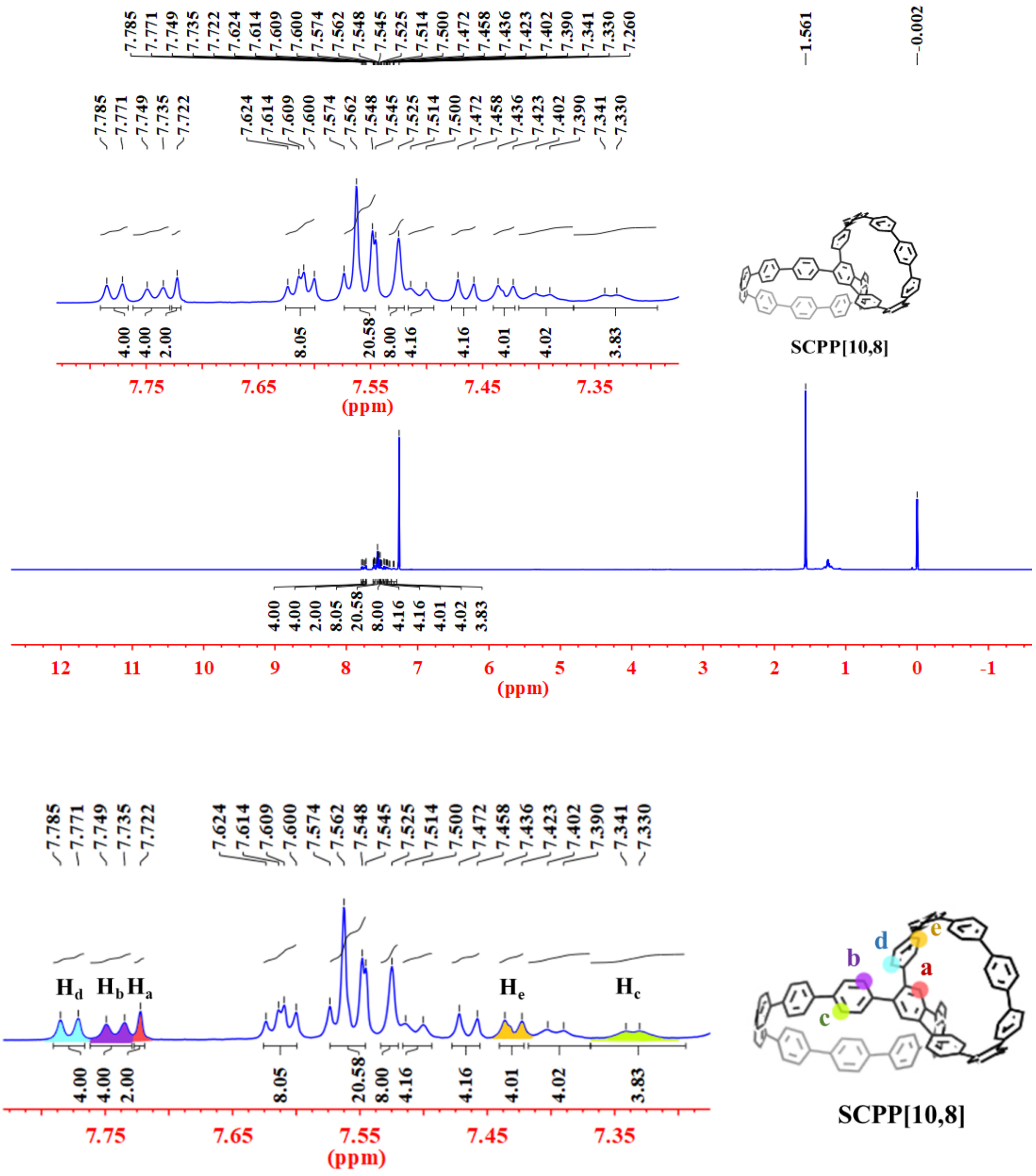

Supplementary Figure 7. ^{1}H NMR spectrum of **SCPP[10,8]** (600 MHz, $CDCl_3$).

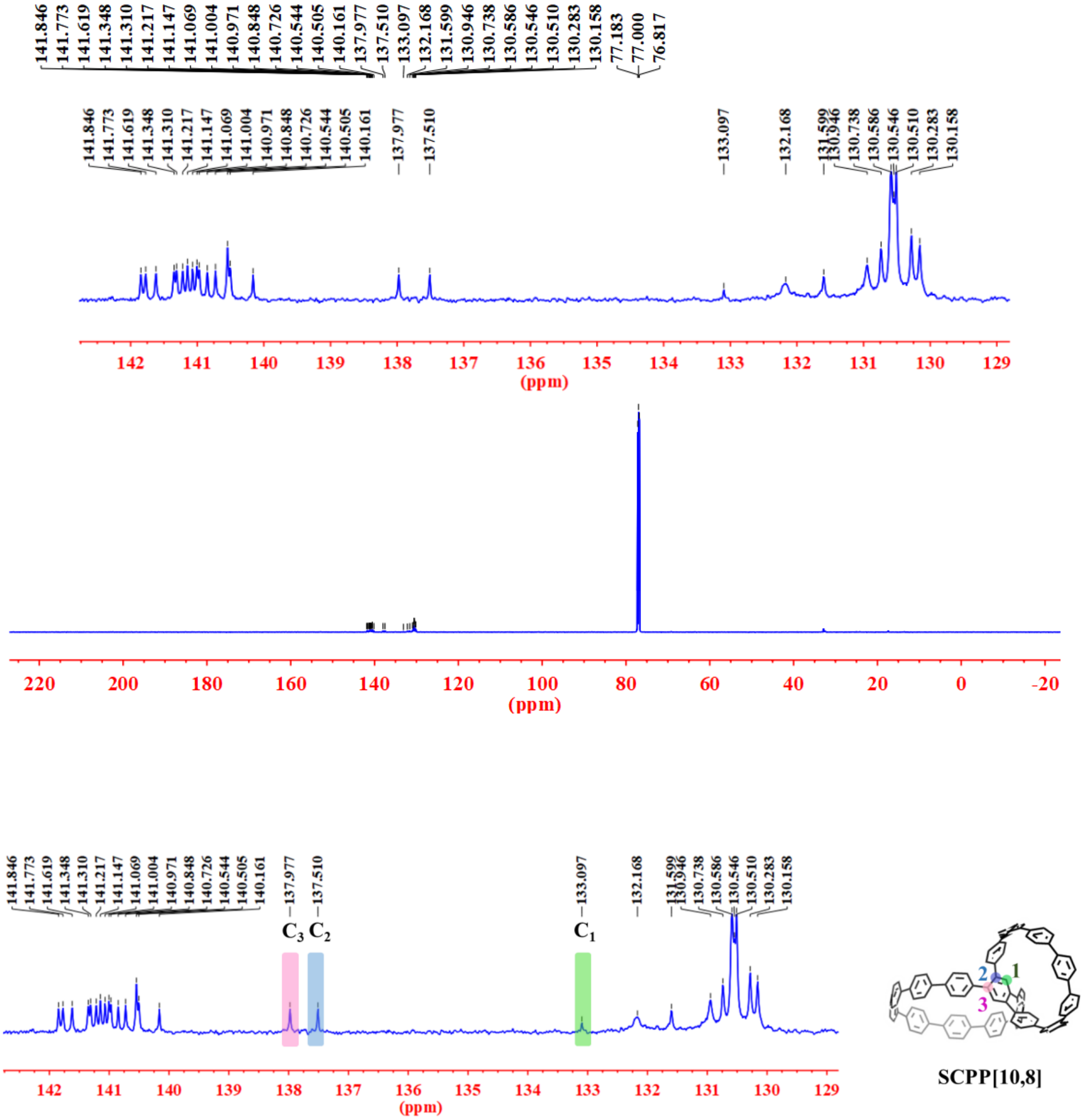

Supplementary Figure 8. ^{13}C NMR spectrum of **SCPP[10,8]** (150 MHz, $CDCl_3$).

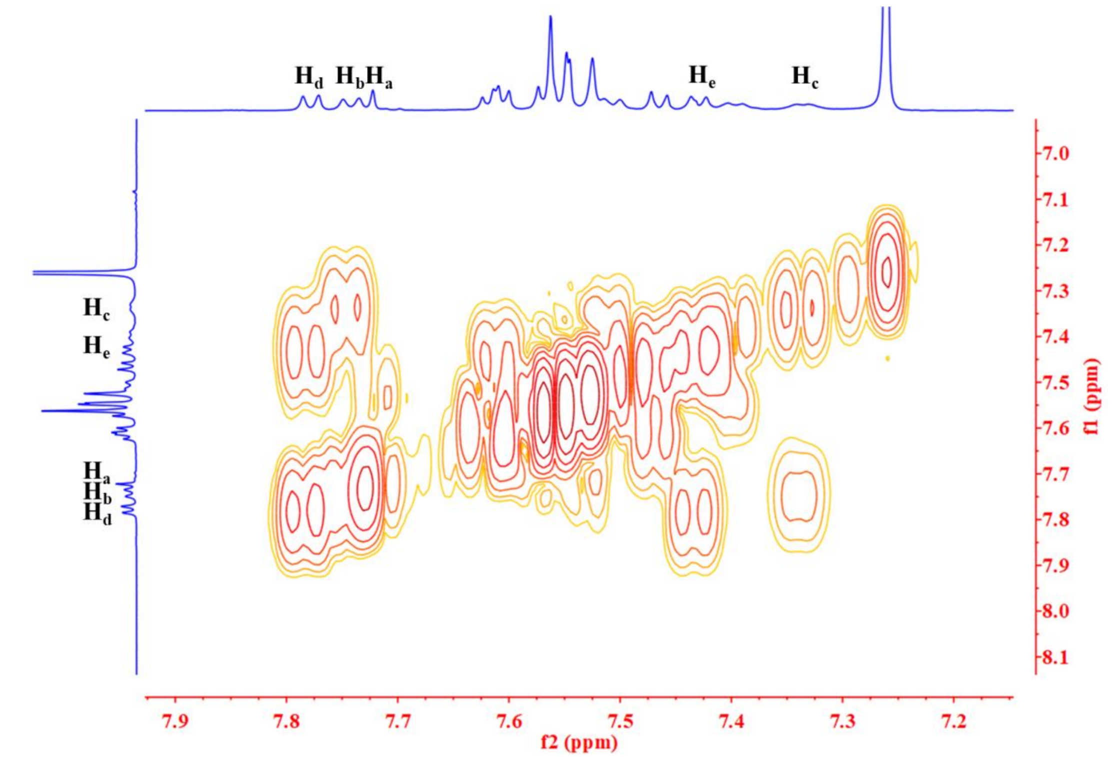

Supplementary Figure 9. Expanded 2D ^1H-^1H COSY NMR spectrum (600 MHz, $CDCl_3$) of **SCPP[10,8]**.

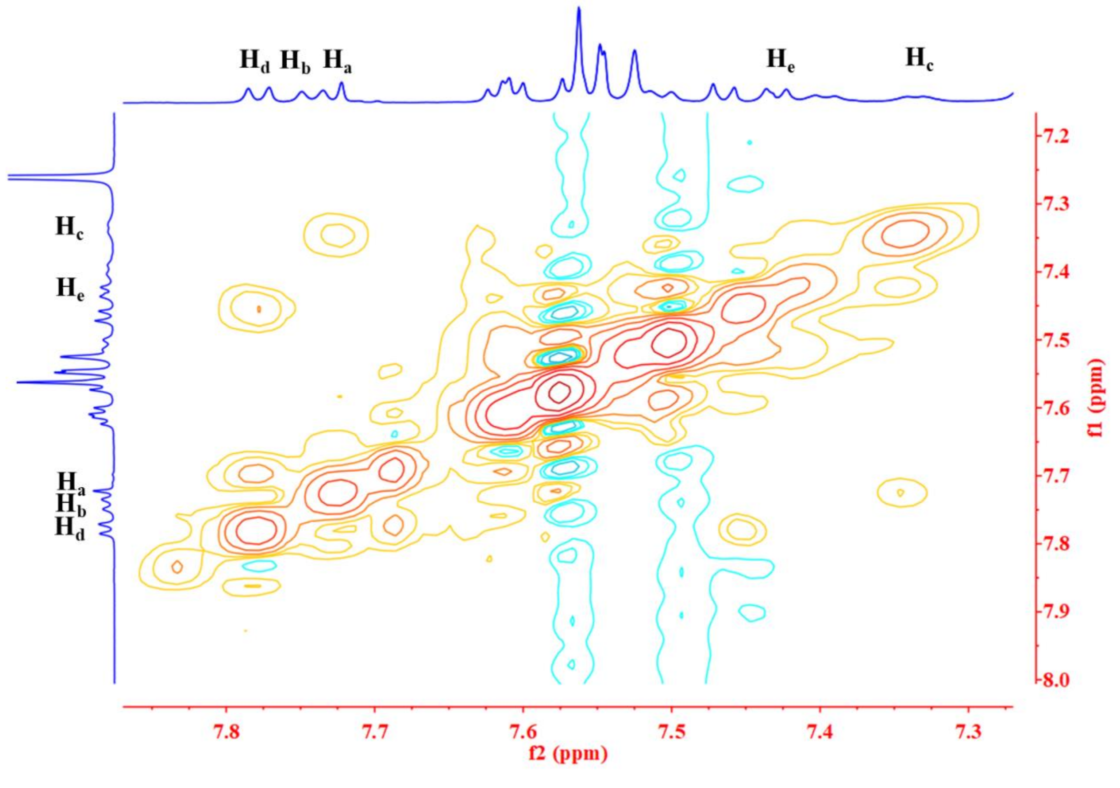

Supplementary Figure 10. Expanded 2D ^1H-^1H NOESY NMR spectrum (600 MHz, $CDCl_3$) of **SCPP[10,8]**.

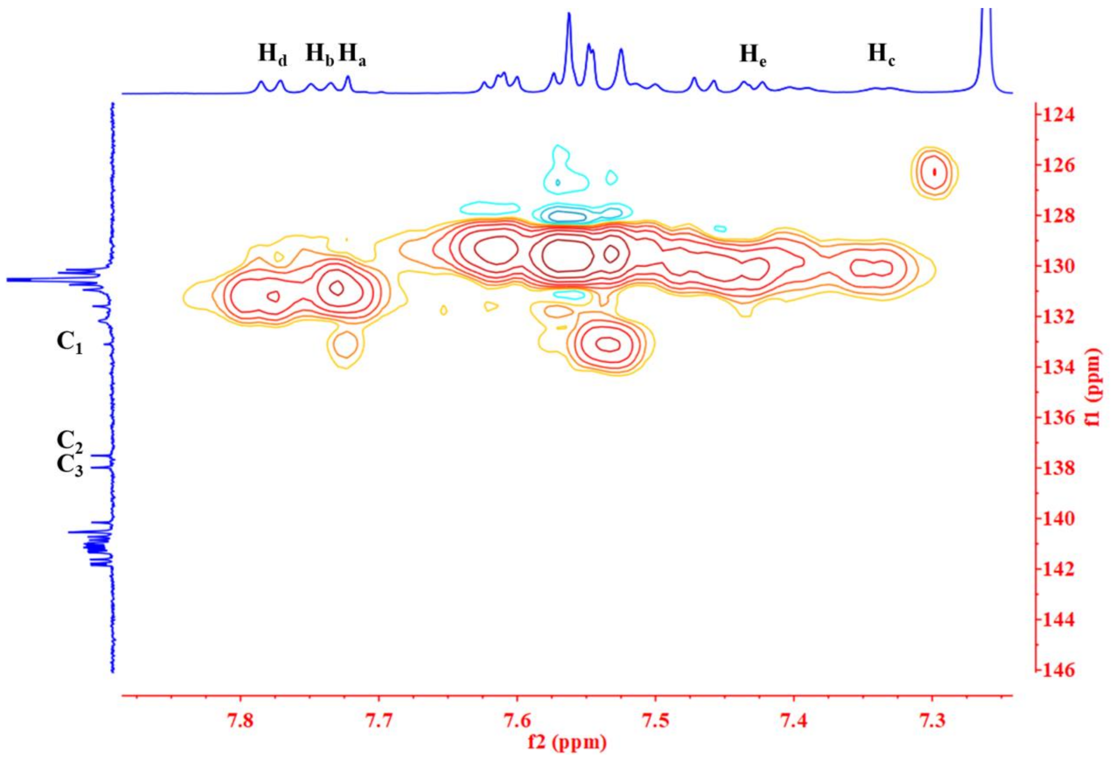

Supplementary Figure 11. Expanded 2D (H, C)-HSQC NMR spectrum (600 MHz, $CDCl_3$) of **SCPP[10,8]**.

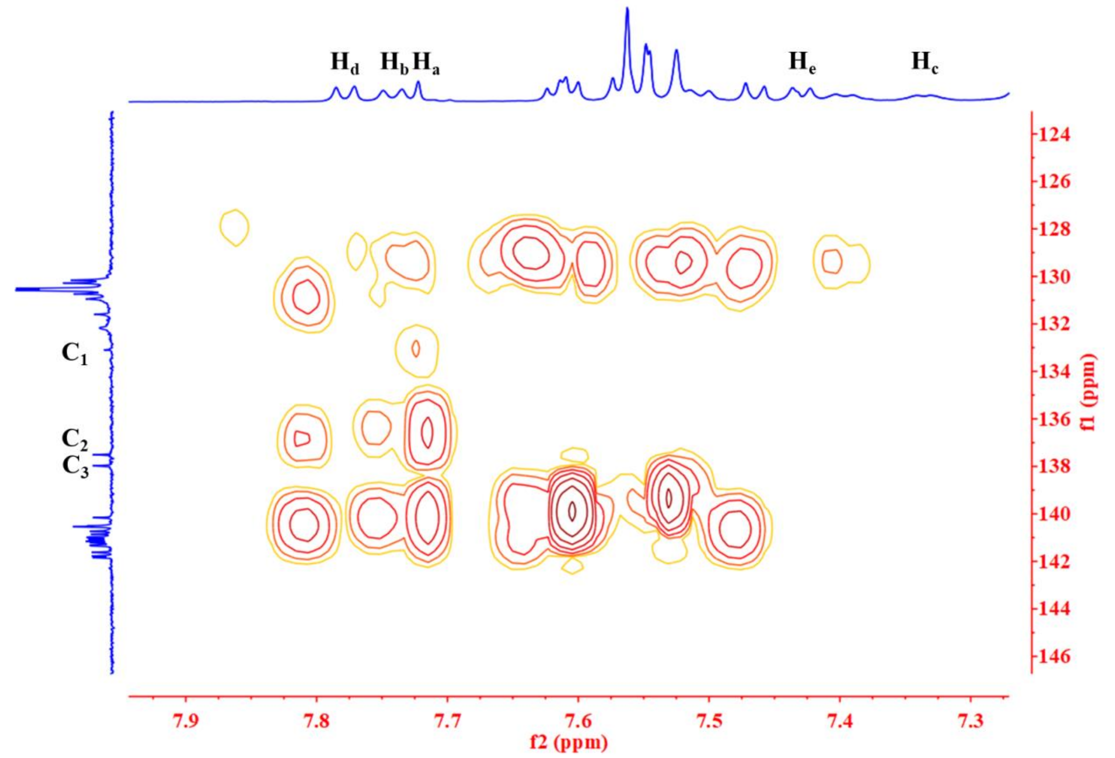

Supplementary Figure 12. Expanded 2D (H, C)-HMBC NMR spectrum (600 MHz, $CDCl_3$) of **SCPP[10,8]**.

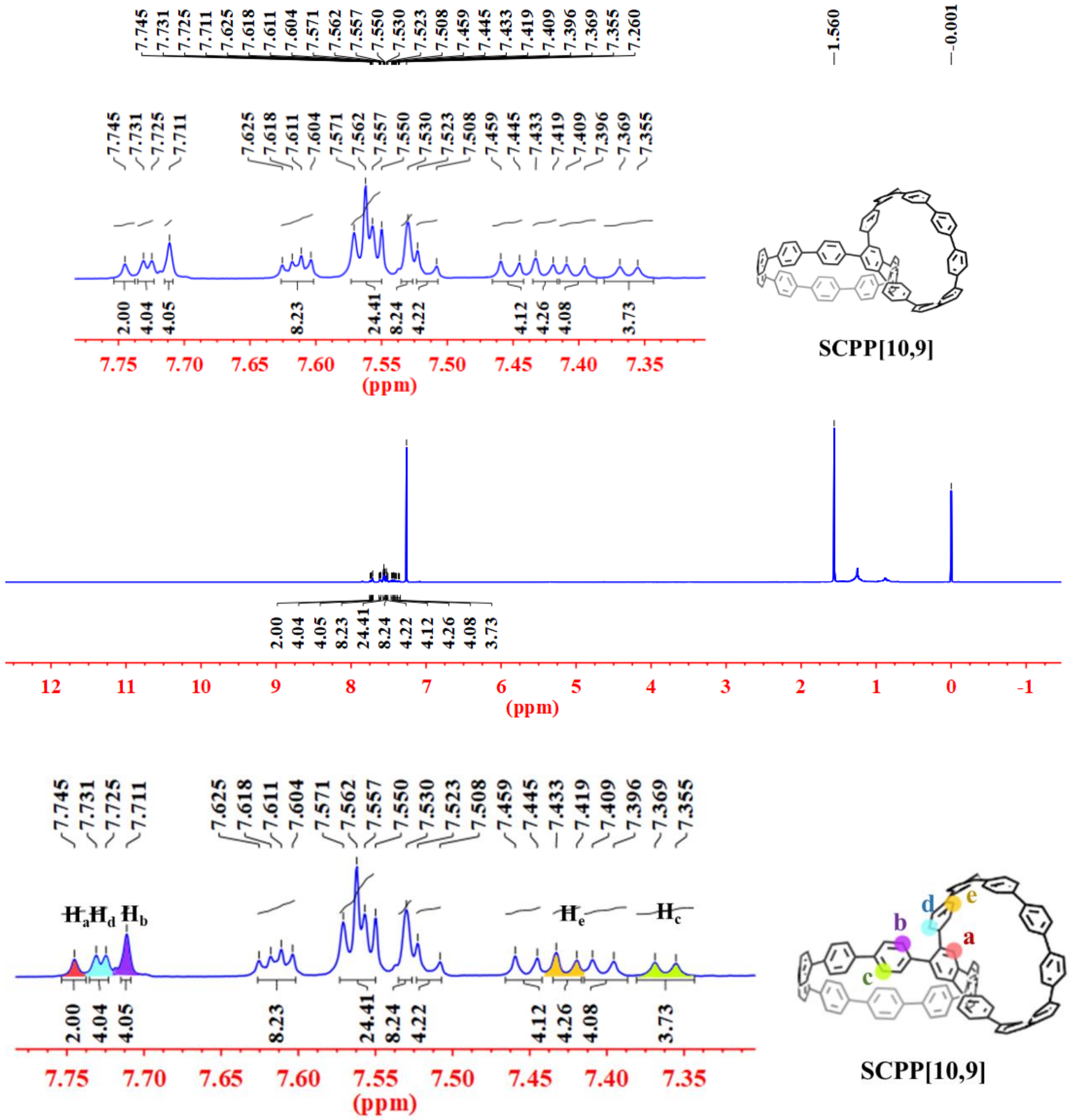

Supplementary Figure 13. ^{1}H NMR spectrum of **SCPP[10,9]** (600 MHz, $CDCl_3$).

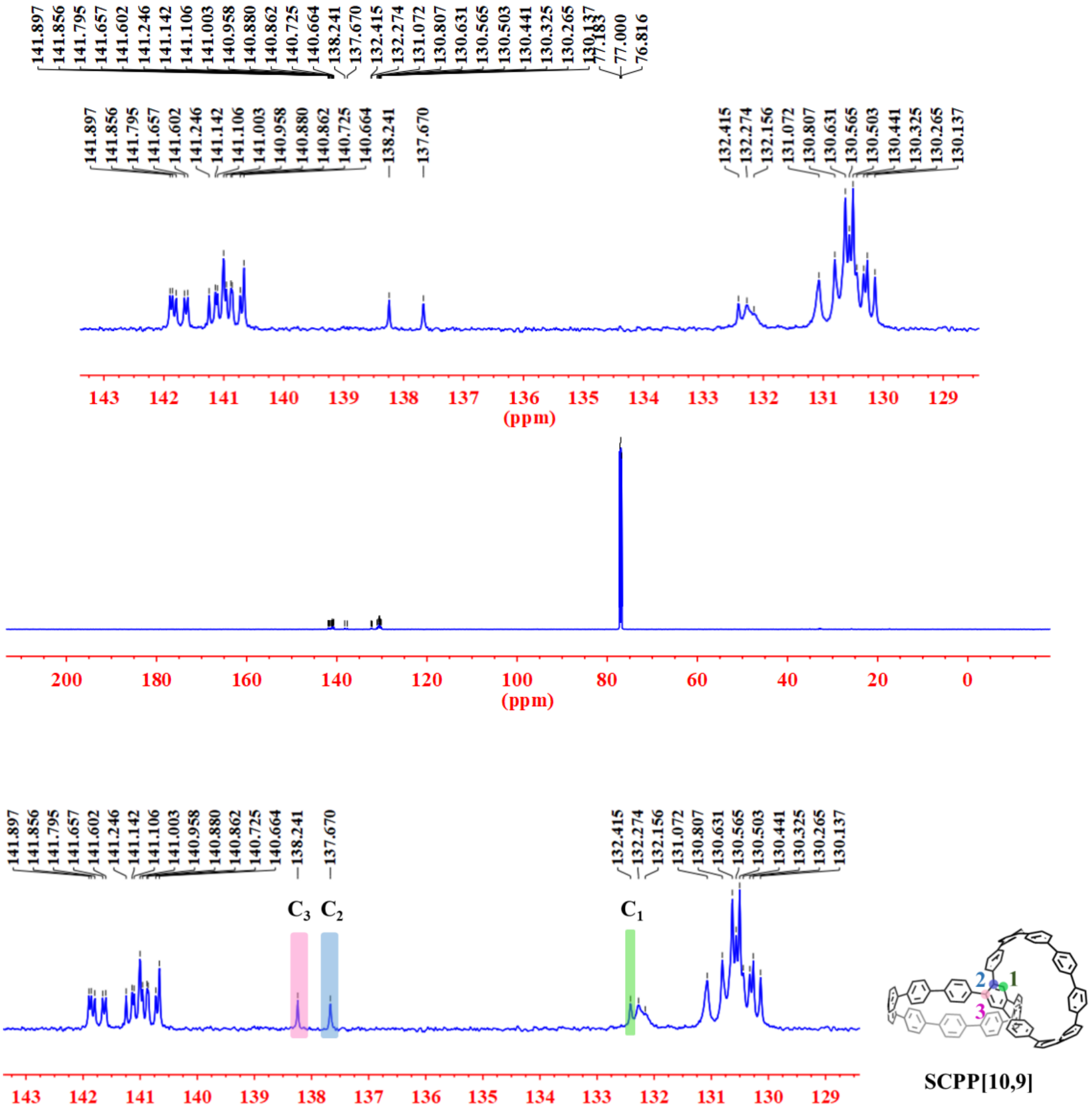

Supplementary Figure 14. ^{13}C NMR spectrum of **SCPP[10,9]** (150 MHz, $CDCl_3$).

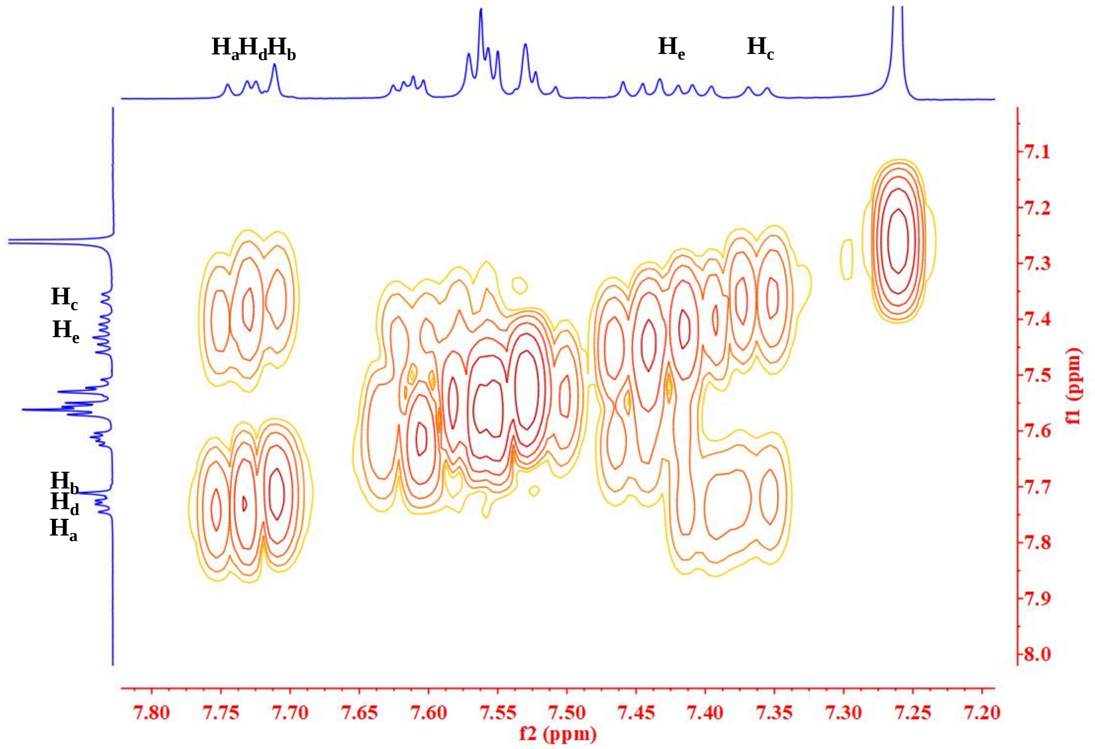

Supplementary Figure 15. Expanded 2D ^{1}H-^{1}H COSY NMR spectrum (600 MHz, $CDCl_3$) of **SCPP[10,9]**.

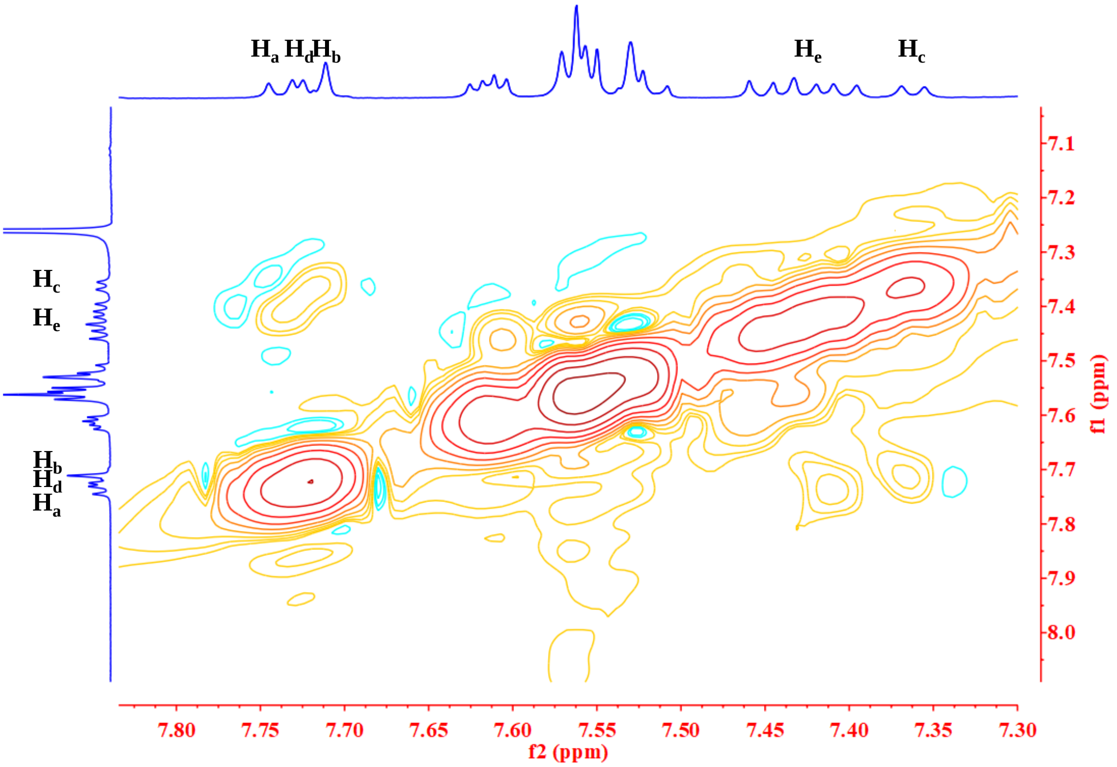

Supplementary Figure 16. Expanded 2D ^{1}H-^{1}H NOESY NMR spectrum (600 MHz, $CDCl_3$) of **SCPP[10,9]**.

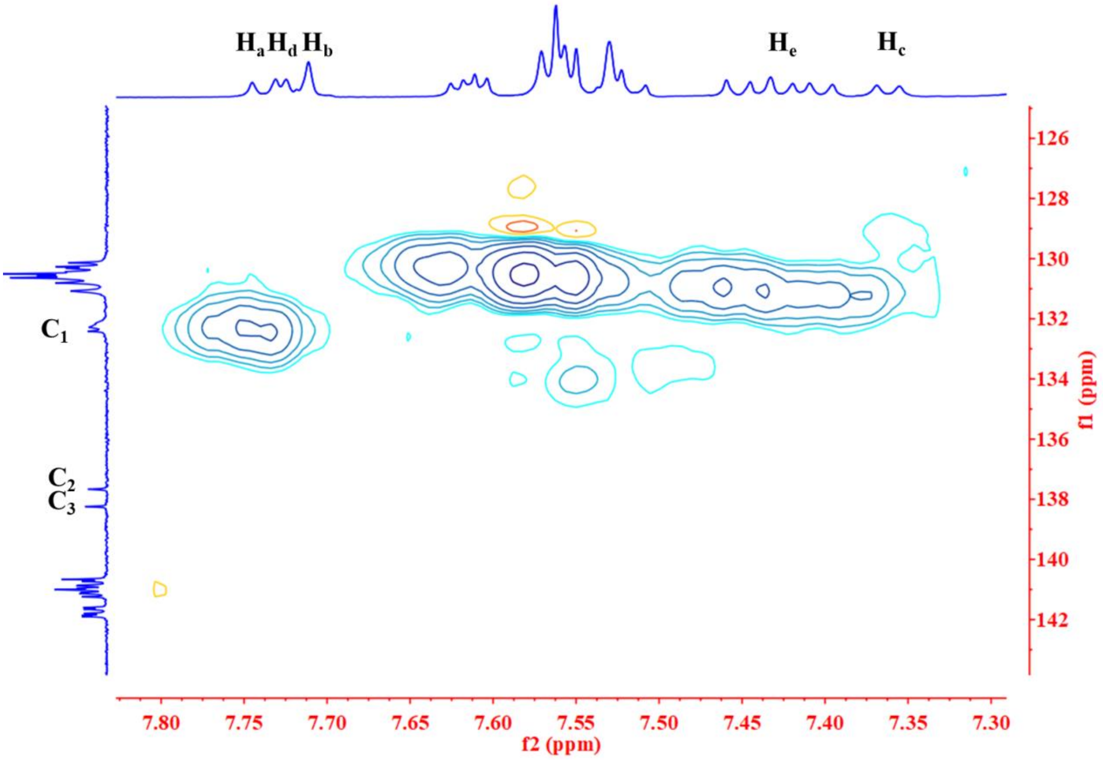

Supplementary Figure 17. Expanded 2D (H, C)-HSQC NMR spectrum (600 MHz, $CDCl_3$) of **SCPP[10,9]**.

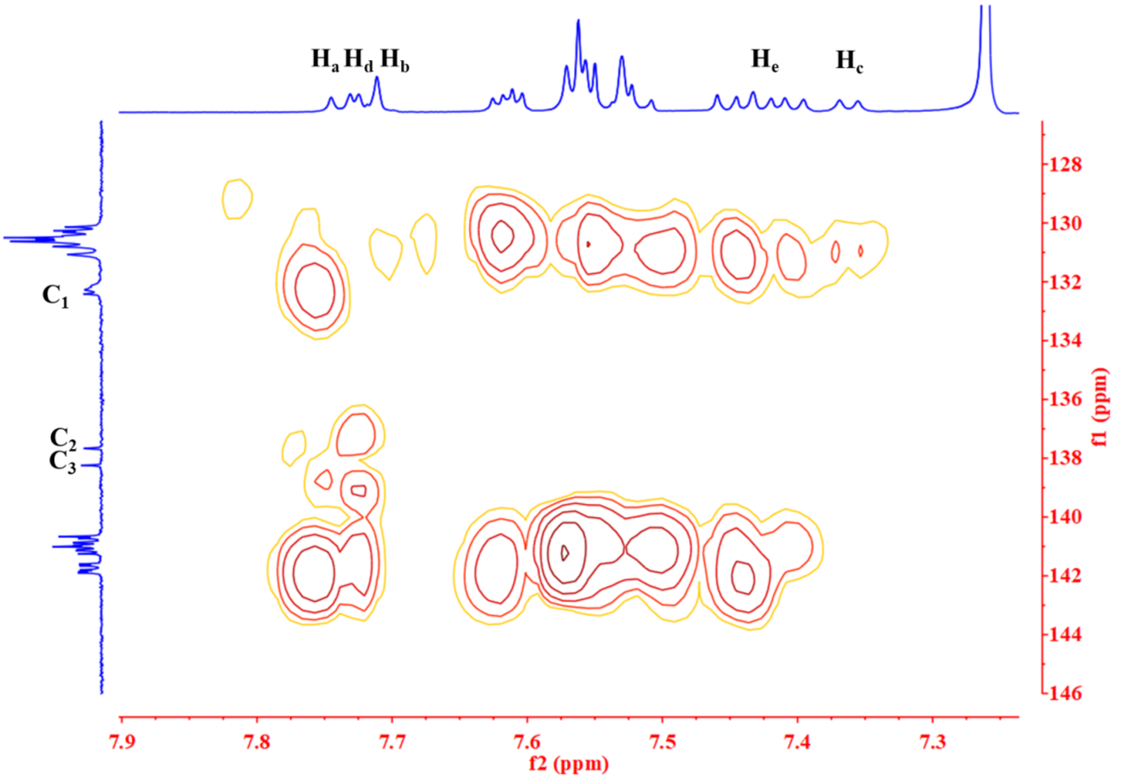

Supplementary Figure 18. Expanded 2D (H, C)-HMBC NMR spectrum (600 MHz, $CDCl_3$) of **SCPP[10,9]**.

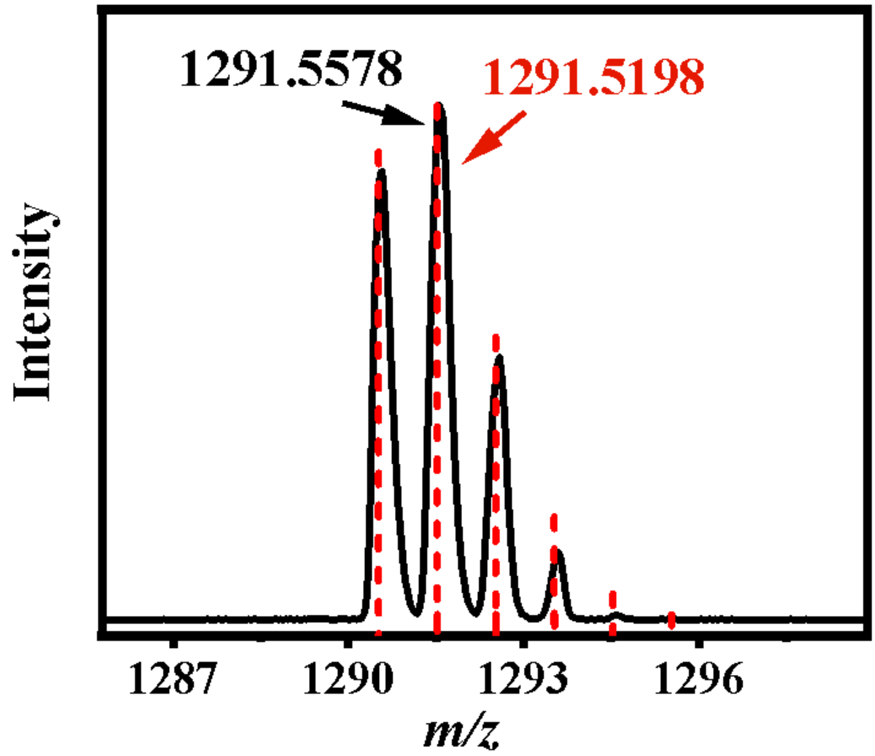

Supplementary Figure 19. MALDI-TOF-MS spectrum (black solid line) and simulated data (red dash line) for **SCPP[10,8]**.

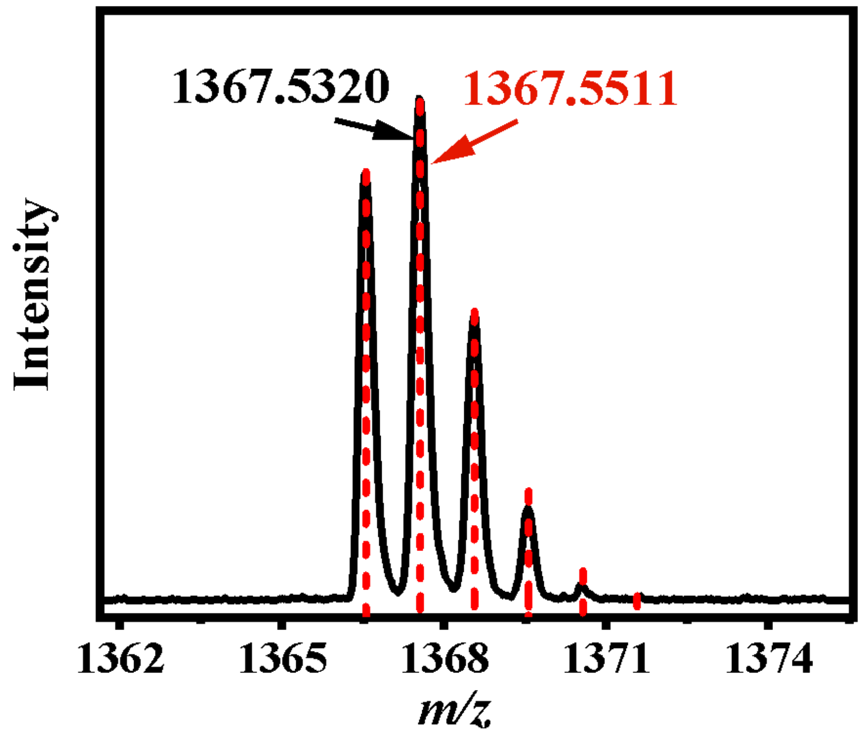

Supplementary Figure 20. MALDI-TOF-MS spectrum (black solid line) and simulated data (red dash line) for **SCPP[10,9]**.

Photophysical Properties of Solids

Supplementary Table 1. The quantum yields of emissions for the different emission processes of **SCPP**s.

Sample	Φ_{S2} [%]	Φ_{S1}[%]
**SCPP[10]**	0.30	3.1
**SCPP[10,9]**	0.12	1.94
**SCPP[10,8]**	0.57	1.20

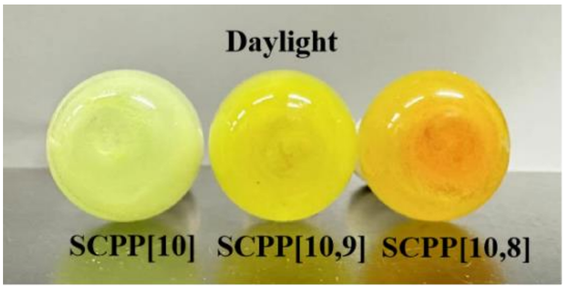

Supplementary Figure 21. The colors of **SCPP[10]**, **SCPP[10,9]**, and **SCPP[10,8]** under daylight.

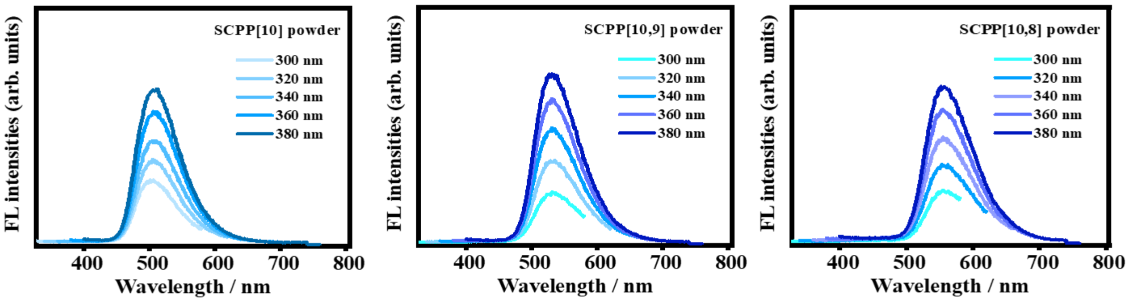

Supplementary Figure 22. Emission spectra of **SCPP**s powder under different excitations.

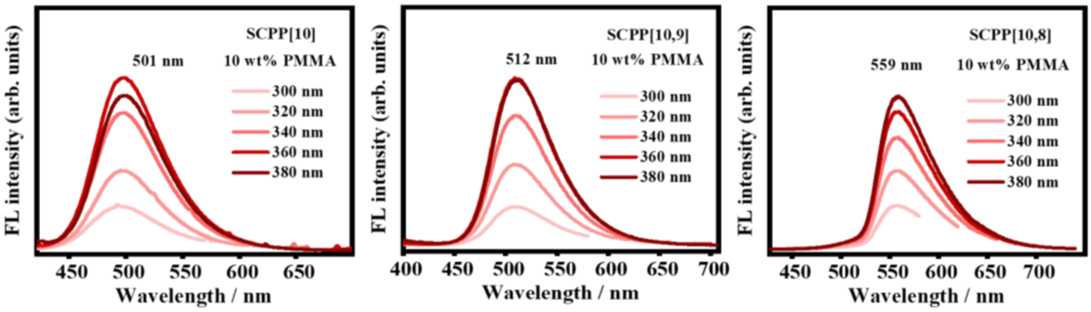

Supplementary Figure 23. Emission spectra of **SCPP**s PMMA films under different excitations.

Theoretical Calculation of Strain Energy

The strain energy of **SCPP[n,m]** and CPP[n] is typically evaluated following the formula:

SE(CPP[n]) = E(CPP[n]) + n × E(Diphenyl) - n × E(1,4-diphenylbenzene) **Eq(1)**

SE(**SCPP[n,m]**) = E(**SCPP[n,m]**) + (n+m) × E(Diphenyl) - (n+m-2×E(1,4-diphenylbenzene) - E(1,2,4,5-tetraphenylbenzene) **Eq(2)**

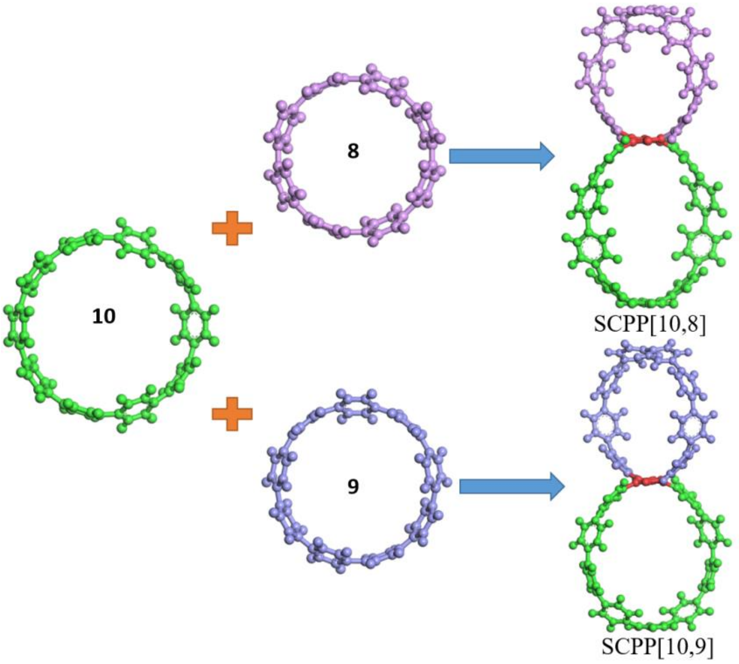

Supplementary Figure 24. The structure of **SCPP[10,8]** and **SCPP[10,9]**.

Supplementary Table 2. Energy (Ha.) for strain energy calculations of **SCPP[10,8]** and **SCPP[10,9]** under the theoretical level of B3LYP-D3BJ/6-31G(d,p)/PCM

Label	Value
Diphenyl	-463.370264
1,4-diphenylbenzene	-694.4617282
1,2,4,5-tetraphenylbenzene	-1156.640227
**SCPP[10,8]**	-3927.154389
strain energy (kcal/mol) of **SCPP[10,8]**	130.98
**SCPP[10,9]**	-4158.265026
strain energy (kcal/mol) of **SCPP[10,9]**	118.95

CPP[10]	-2310.818035
strain energy (kcal/mol) of CPP[10][§]	60.62
CPP[9]	-2079.71604
strain energy (kcal/mol) of CPP[9]	67.23
CPP[8]	-1848.613802
strain energy (kcal/mol) of CPP[8]	73.99

Frontier Molecular Orbitals

Supplementary Table 3. Energy level (unit: eV) of frontier molecular orbitals for **SCPP[10,8]** and **SCPP[10,9]** under the theoretical level of B3LYP-D3BJ/6-31G(d,p)/PCM (tetrahydrofuran)

Molecule	SCPP[10,8]	SCPP[10,9]
LUMO+3	-1.46969	-1.40847
LUMO+2	-1.52466	-1.53500
LUMO+1	-1.77392	-1.70834
LUMO	-2.07733	-2.01637
HOMO	-4.97428	-5.07986
HOMO-1	-5.22326	-5.29238
HOMO-2	-5.48667	-5.47415
HOMO-3	-5.50000	-5.55905
E(HOMO-LUMO)	-2.89694	-3.06348

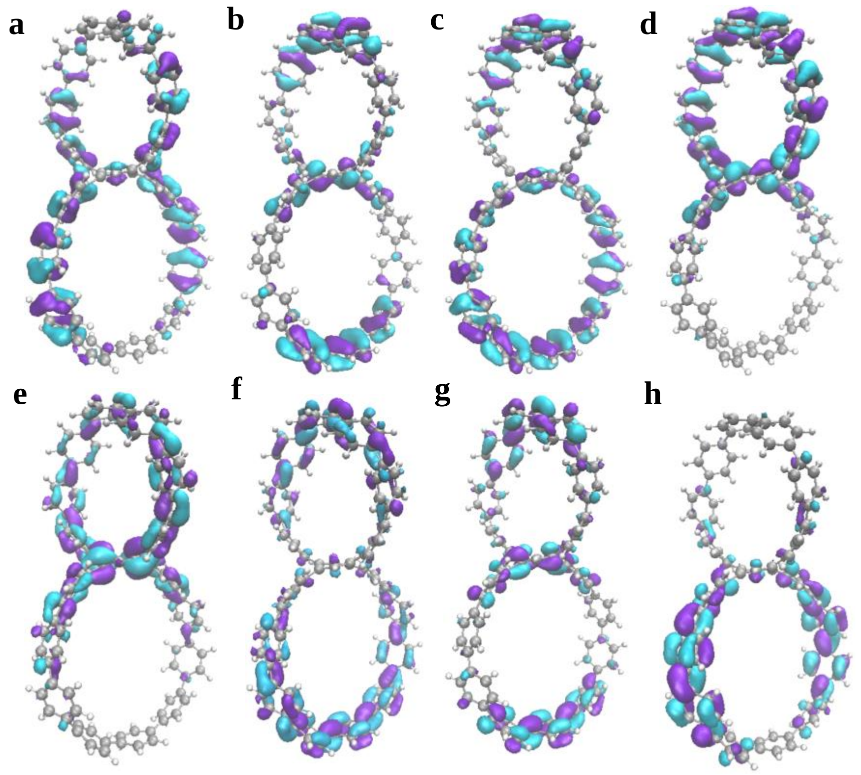

Supplementary Figure 25. Frontier molecular orbitals of **SCPP[10,8]**: HOMO-3 (**a**), HOMO-2 (**b**), HOMO-1 (**c**), HOMO (**d**), LUMO (**e**), LUMO+1 (**f**), LUMO+2 (**g**) and LUMO+3 (**h**).

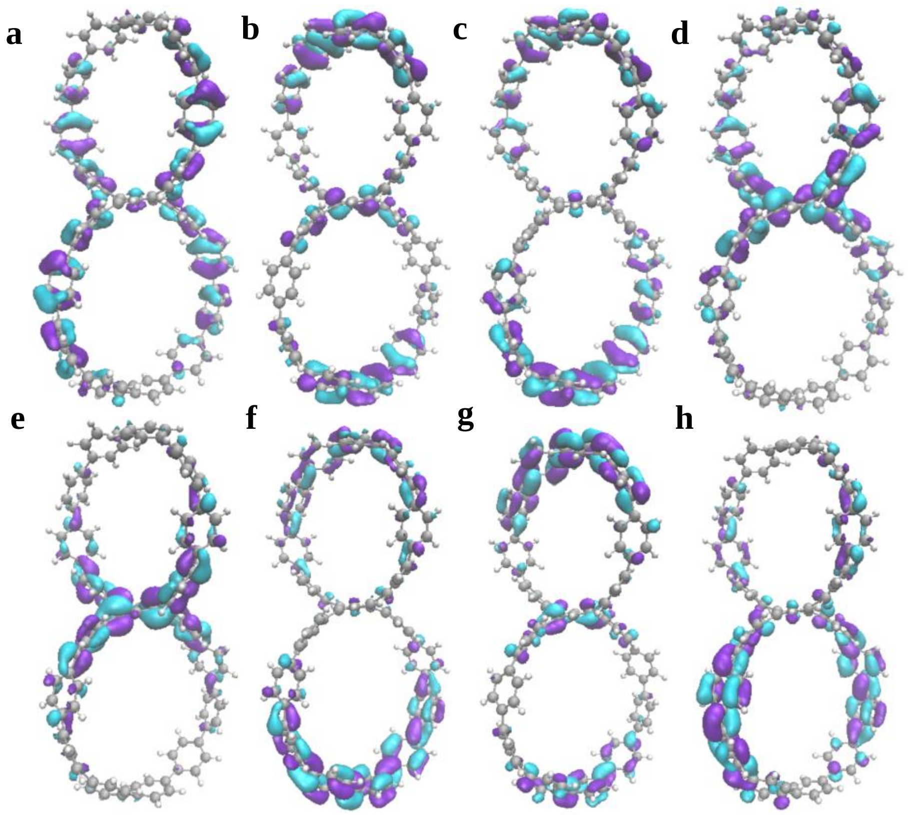

Supplementary Figure 26. Frontier molecular orbitals of **SCPP[10,9]**: HOMO-3 (**a**), HOMO-2 (**b**), HOMO-1 (**c**), HOMO (**d**), LUMO (**e**), LUMO+1 (**f**), LUMO+2 (**g**) and LUMO+3 (**h**).

DFT Calculated Oscillator Strengths and Transitions

Supplementary Table 4. TD-DFT calculated oscillator strengths (f_{osc} > 0.01) and transitions of **SCPP[10,8]** from the level of CAM-B3LYP/6-31G*.

Excited state	Energy (eV)	Wavelength (nm)	Osc. Strength	Major contributions
1	3.1120	398.41	0.06710	H → L (68.5%)
2	3.4429	360.12	0.07400	H-1 → L (28.6%)<br>H → L+1 (16.2%)
3	3.7272	332.65	0.84370	H-1 → L+1 (27.1%)<br>H → L+2 (19.7%)<br>H-2 → L (18.7%)
4	3.7754	328.40	3.97680	H-3 → L (35.5%)<br>H → L+3 (15.4%)
5	3.8538	321.72	0.38850	H-5 → L (26.8%)
6	4.0886	303.24	0.84200	H-4 → L (13.1%)<br>H-1 → L+3 (12.9%)
7	4.1167	301.17	1.19190	H → L+5 (20.6%)
8	4.4382	279.36	0.33140	H-3 → L+2 (17.9%)<br>H-3 → L (15.0%)
10	4.5286	273.78	0.02340	H → L+3 (19.2%)

Supplementary Table 5. TD-DFT calculated oscillator strengths (f_{osc} > 0.01) and transitions of **SCPP[10,9]** from the level of CAM-B3LYP/6-31G*.

Excited state	Energy (eV)	Wavelength (nm)	Osc. Strength	Major contributions
1	3.2417	382.47	0.14670	H → L (64.8%)
3	3.7768	328.28	4.45440	H-3 → L (38.2%)<br>H → L+3 (23.6%)
4	3.7836	327.69	1.00030	H-1 → L+1 (27.0%)<br>H-2 → L (21.6%)<br>H → L+2 (18.1%)
5	3.9233	316.02	0.47440	H-5 → L (27.7%)
6	4.0895	303.18	0.02360	H-4 → L (20.7%)<br>H → L+4 (20.0%)<br>H-1 → L+3 (18.5%)<br>H-3 → L+1 (17.7%)

7	4.1270	300.42	1.75480	H → L+5 (29.9%)<br>H-2 → L+1 (20.9%)
8	4.4584	278.09	0.45890	H-3 → L+2 (17.1%)<br>H-7 → L (14.7%)<br>H-3 → L (12.9%)
9	4.4923	275.99	0.38620	H-3 → L+3 (14.1%)<br>H-2 → L+2 (13.5%)<br>H-1 → L+5 (12.4%)
10	4.5813	270.63	0.01030	H → L+3 (28.4%)<br>H-3 → L (17.5%)

Supplementary Table 6. TD-DFT calculated oscillator strengths ($f_{osc} > 0.01$) and transitions of **SCPP[10]** from the level of CAM-B3LYP/6-31G*.

Excited state	Energy (eV)	Wavelength (nm)	Osc. Strength	Major contributions
1	3.3021	375.47	0.12630	H → L (59.6%)
2	3.5112	353.11	0.02330	H-1 → L (25.6%)<br>H → L+1 (19.7%)
3	3.7559	330.11	1.07500	H-1 → L+1 (25.6%)<br>H-2 → L (20.6%)<br>H → L+2 (20.3%)
4	3.8013	326.16	4.75940	H-3 → L (36.5%)<br>H → L+3 (24.2%)
5	3.9371	314.91	1.23430	H-5 → L (34.9%)<br>H-1 → L+2 (16.9%)
7	4.1086	301.77	1.30660	H → L+5 (32.9%)<br>H-2 → L+1 (16.7%)
8	4.4367	279.45	0.30680	H-3 → L+3 (15.0%)<br>H-2 → L+2 (13.3%)
9	4.4537	278.38	0.50450	H-3 → L+2 (18.6%)<br>H-7 → L (16.1%)

TDDFT-calculated Absorption Spectra

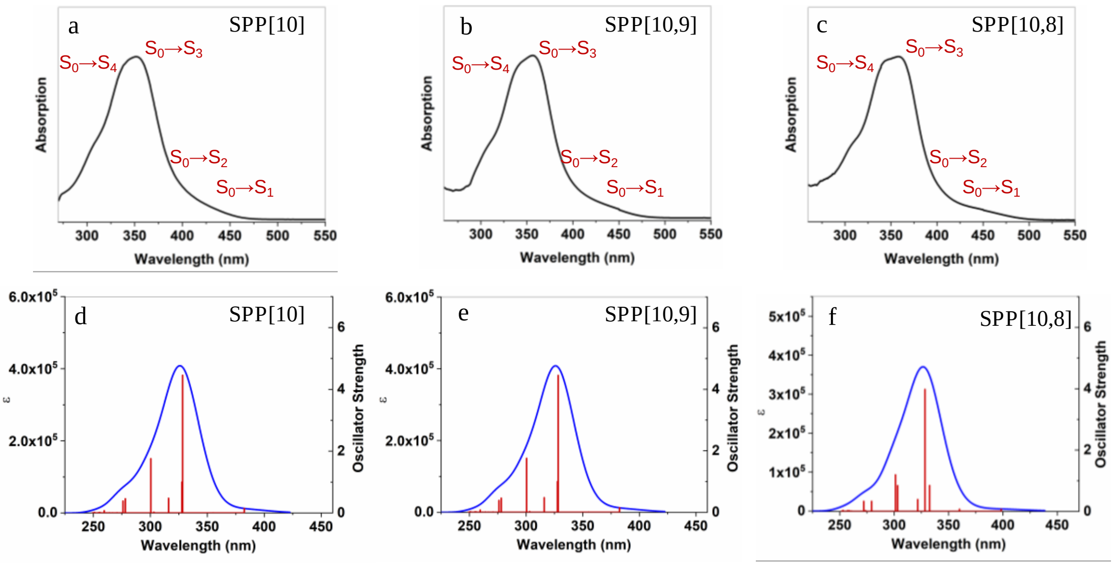

Supplementary Figure 27. TDDFT-calculated absorption spectra of **SCPP[10,8]** (**a**) and (**d**) , **SCPP[10,9]** (**b**) and (**e**) , and **SCPP[10]** (**c**) and (**f**) along with the oscillator strengths.

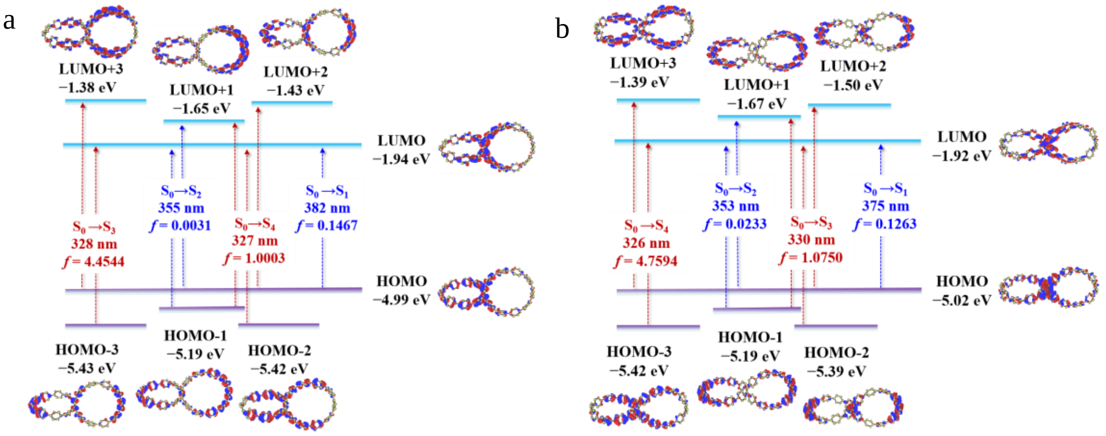

Supplementary Figure 28. TDDFT-calculated molecular orbitals and energy diagrams of **SCPP[10,9]** (**a**) and **SCPP[10]** (**b**). *f* value represents the oscillator strength.

Excited-state Calculations and Diagram of Photophysical Processes

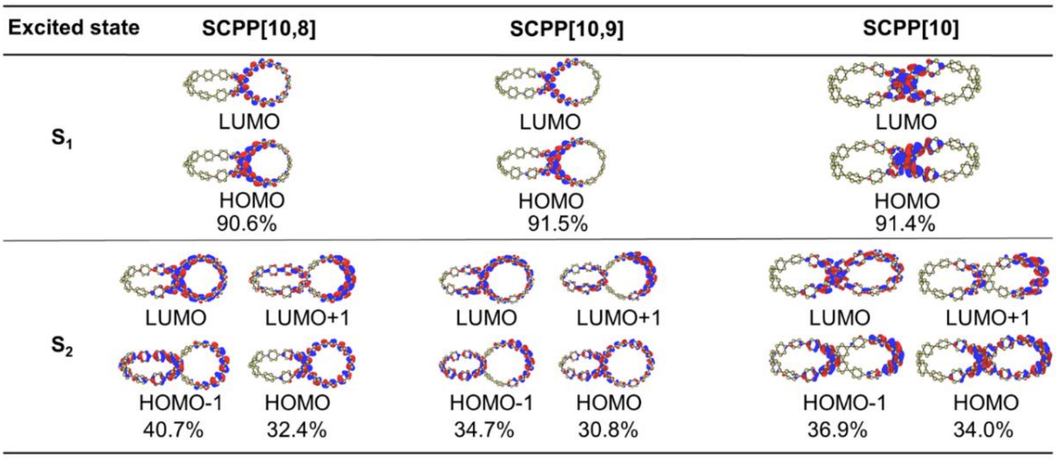

Supplementary Figure 29. The excited-state calculations and the corresponding molecular orbitals.

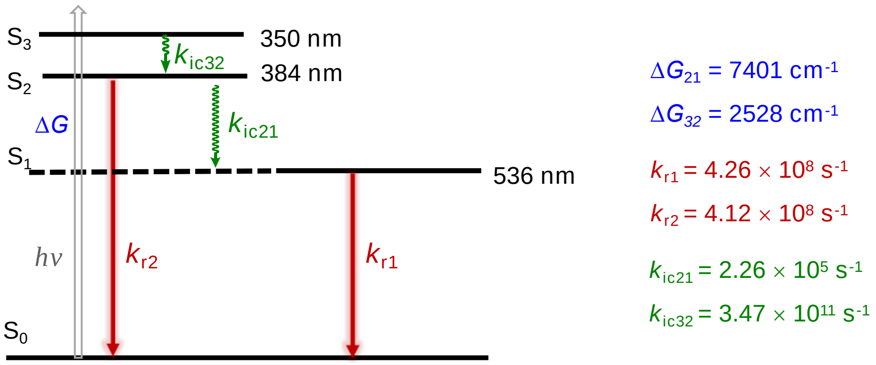

Supplementary Figure 30. Jablonski diagram showing the calculated photophysical processes of **SCPP[10,9]**. k_{ic}: internal conversion rate, k_r: radiative transition rate, ΔG: energy gap between two excited states.

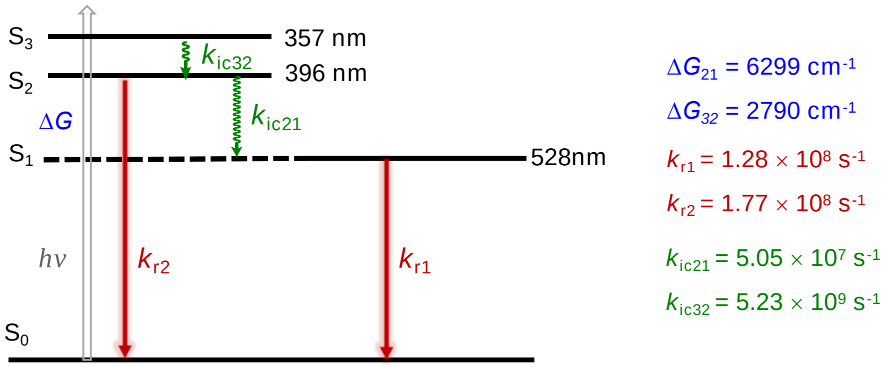

Supplementary Figure 31. Jablonski diagram showing the calculated photophysical processes of **SCPP[10]**. k_{ic}: internal conversion rate, k_r: radiative transition rate, ΔG: energy gap between two excited states.

Emission Lifetimes and Fitting Results

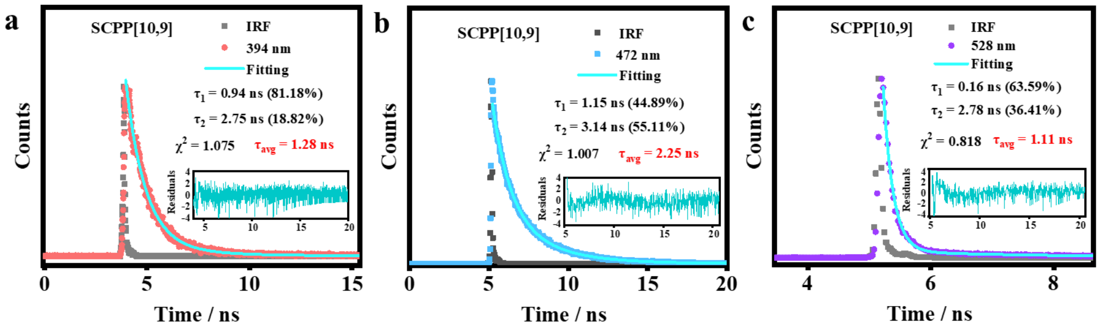

Supplementary Figure 32. Emission lifetimes for **SCPP[10,9]** at 394 nm (**a**), 472 nm (**b**), and 528 nm (**c**).

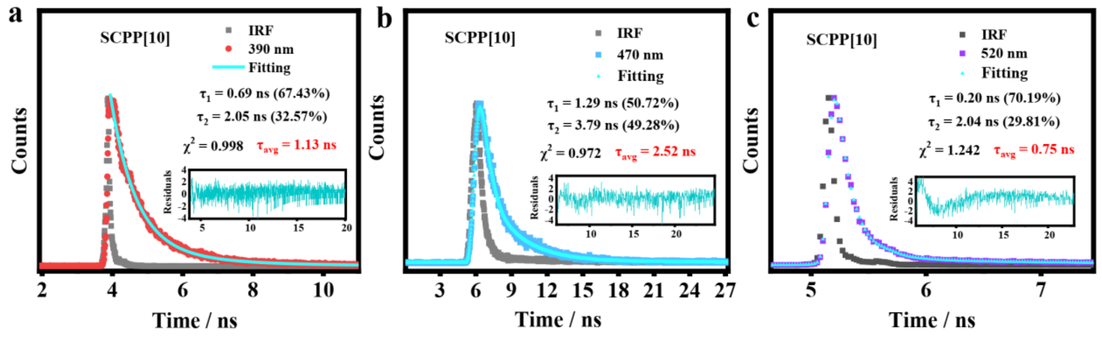

Supplementary Figure 33. Emission lifetimes for **SCPP[10]** at 390 nm (**a**), 470 nm (**b**), and 520 nm (**c**).

Supplementary Table 7. Biexponential fitting results of PL decays for **SCPPs**.

Sample	wavelength	τ_1 (10^{-9} s)	τ_2 (10^{-9} s)	A_1 (%)	A_2 (%)	τ_{avg} (10^{-9} s)
**SCPP[10]**	390 nm	0.69	2.05	67.43	42.57	1.13
	470 nm	1.29	3.79	50.72	49.28	2.52
	520 nm	0.20	2.04	70.19	29.81	0.75
**SCPP[10,9]**	394 nm	0.94	2.75	81.18	18.82	1.28
	472 nm	1.15	3.14	44.89	55.11	2.25
	528 nm	0.16	2.78	63.59	36.41	1.11
**SCPP[10,8]**	392 nm	0.80	2.38	91.78	8.22	0.93
	467 nm	0.69	2.96	21.36	78.64	2.48
	545 nm	0.21	3.51	61.74	38.26	1.47
